# European health research and globalisation: is the public-private balance right?

**DOI:** 10.1186/1744-8603-7-5

**Published:** 2011-03-22

**Authors:** Mark McCarthy

**Affiliations:** 1 Professor of Public Health, Department of Epidemiology and Public Health, University College London, 1-19 Torrington Place, London WC1E 6BT, UK

## Abstract

**Background:**

The creation and exchange of knowledge between cultures has benefited world development for many years. The European Union now puts research and innovation at the front of its economic strategy. In the health field, biomedical research, which benefits the pharmaceutical and biotechnology industries, has been well supported, but much less emphasis has been given to public health and health systems research. A similar picture is emerging in European support for globalisation and health

**Case studies:**

Two case-studies illustrate the links of European support in global health research with industry and biomedicine. The European Commission's directorates for (respectively) Health, Development and Research held an international conference in Brussels in June 2010. Two of six thematic sessions related to research: one was solely concerned with drug development and the protection of intellectual property. Two European Union-supported health research projects in India show a similar trend. The Euro-India Research Centre was created to support India's participation in EU research programmes, but almost all of the health research projects have been in biotechnology. New INDIGO, a network led by the French national research agency CNRS, has chosen 'Biotechnology and Health' and funded projects only within three laboratory sciences.

**Discussion:**

Research for commerce supports only one side of economic development. Innovative technologies can be social as well as physical, and be as likely to benefit society and the economy. Global health research agendas to meet the Millenium goals need to prioritise prevention and service delivery. Public interest can be voiced through civil society organisations, able to support social research and public-health interventions. Money for health research comes from public budgets, or indirectly through healthcare costs. European 'Science in Society' programme contrasts research for 'economy', using technical solutions, commercialisation and a passive consumer voice for civil society, compared with research valuing 'collectivity', organisational and social innovations, open use, and public accountability.

**Conclusions:**

European policy currently prioritises health research in support of industry. European institutions and national governments must also support research and innovation in health and social systems, and promote civil society participation, to meet the challenges of globalisation.

## Introduction

This paper is one in a series of papers in *Globalisation and Health *following the seminar 'Health systems, health economies and globalisation: social science perspectives' held at the London School of Economics in July 2010 with participants jointly from UK and India. It asks, from a European and global perspective, what knowledge will best promote health. The Background presents a historical example of the globalisation of knowledge. The European Perspectives section describes development of the European 'knowledge-based economy', policies and structures for research, and the position of health research. Two Case-study examples follow, of European engagement with globalisation and health in India. The Discussion considers the implications for health of for-profit research, the role of civil society organisations, and the contribution that social sciences can give to globalisation and public health.

## Background

### Globalisation of knowledge

The expansion of trade, communication and travel that is implied in the term globalisation has been a gradual process over past centuries, but with increasing speed and impact into the present century. Ideas and knowledge are significant features within this process, controlling both development within countries and also available for exchange and trade themselves. A remarkable example from a European perspective is described by Menzies [1] in a controversial book entitled '1434'. He suggests that arrival in Venice of ships from China in that year, originating from a grand fleet sent westwards to demonstrate China's power and advanced culture to the world, contributed substantially to globalisation through the diffusion of knowledge. Menzies contends that the technologies developed and well known to the Chinese suddenly emerged in renaissance Italy. He shows that the drawings of technologies across a wide range of fields, from canal locks, to winches, to helicopters, by Leonardo da Vinci had been printed in practical books circulating in China a century previously. Shortly after, with changing politics at home, the east closed its doors to the west But the new technologies fed into the reformation and Europe's industrial development. Now, in the electronic era, leadership in technology that moved from Europe to America in the twentieth century is again ranging across the whole world.

Science, technology and innovation are important drivers of economic change, although innovation is rarely instant and older technologies continue both in the world and within countries for long periods in the face of alternatives that may be cheaper, speedier or less polluting [2]. New methods of production, new products and new social organisations can create competitive advantage [3] that leads to economic advancement - the aim now of almost all political systems. Science is neutral but its effects can be political [4], enabling wars as well as wealth [5], and indeed the pressures of war have also led to new technologies. The direction of science towards humanitarian ends is particularly demonstrated in health science, but the underlying purpose of knowledge for (here social) development is the same. Since science and technology produces wealth, politicians want it.

The challenge for creative scientists is to direct knowledge across the full range of cultural development. 'Technologies' can be social as well as mechanical. One of the recognised innovations in the UK during the Second World War was 'operational research', in which scientific systems of thinking (especially mathematics) were applied to real-world problem-solving [6]. Similarly, the 1942 Beveridge Report, setting out a new system for social justice in Britain, also resulted from collective pressures of a war which impacted not just on forces overseas but also on the civil population at home. Now in the health field, social innovations in the organisation of services and care, and in the prevention of disease through changing behaviours and social determinants, are creating new ways of understanding and controlling both the physical and the social worlds.

## European Perspectives

### Science in Europe

The priority of invention and achievements in science by China before the European renaissance were established by Joseph Needham [7]. India's scientific achievements are less well researched, although steel was an early invention, as shown in the rust-free iron pillar in Delhi dated 402CE [8]. Europe has been at the forefront of science and technology in the recent past, and wishes to be so in the future. In contrast to the Imperial model, however, Europe - developing from city-republics [9] - inclines to a decentralised competitive model. Towns, regions and countries compete with each other; individuals compete, and use legal patents to own exclusive rights for intellectual property; and now universities, the contemporary knowledge institutions, compete to attact students for income and faculty members to promote research ratings and enhance prestige.

The European Union now includes most of the countries of geographical Europe. It remains relatively weak at national level, as the member states retain the main levers of economic control, and the European Union's own budget is only 1% of the total European GNP. Yet the European Union has two great strengths: it is a framework for international collaboration that is increasingly accepted and welcomed by its citizens; and it holds, in its legal directives, the means for long-term regulation and convergence of economic and social practices. Implementing the laws required of the European *aquis communautaire *has been a major factor in transforming the former communist states of Eastern Europe.

The European Union has three main structures: the Council of Ministers - the political heads of member states, approving laws; the European Parliament - directly elected parliamentarians debating policies; and the European Commission - the administration, holding both budget and bureaucracy and therefore executive power. The Commission has 'directorates', each headed by a Commissioner, similar to ministries in member states. Science was a field for collaboration relatively early in the European Community (the antecedent of the European Union). In the 1970 s, the directorate for research developed programmes initiating cooperation between European academics. It offered grants for travel and meetings, as well as supporting some larger institutes (eg CERN) to bring European scientist together on one campus. From the perspective of European Community legal competence, biomedical research was accepted from the 1970 s as within the field of science; and biomedicine has taken a rising proportion of the enlarging budget within the Research Framework Programmes [10]. On the other hand 'health' was regarded as outside European competence until the 1992 Treaty of Maastricht. This thinking, that biomedicine 'science' is within DG Research, while public health and health systems are separate within DG Health - and without a strong research perspective - has persisted to the present.

The European Union's Lisbon Strategy in 2000 proposed that Europe should become the 'leading knowledge-based economy' in the world by 2010 [11]. There should be more funding for research, the knowledge gained should be used to develop new products for competitive international markets, and business should contribute a higher proportion of funds. Yet this hope has not been fulfilled. In 2010, the average for R&D spending in the European Union remained below 2% of total GDP, compared with 2.6% in the US and 3.4% in Japan. This difference is mainly due to less R&D by private companies in Europe [12].

The European Union funds only a small proportion of all science in Europe, which is mostly financed from national resources; and in some areas, European collaboration is not high on the agenda. For example, member states have been cautious in signing up to the European Commission's 'Joint Programming Initiative' which hopes to create common collaborative research programmes [13]. Yet from the view-point of European Commission administrators seeking to expand the science and innovation base in Europe, the research programme is an important instrument for dissemination and economic development, providing technology transfer between collaborative teams and funds for setting up new activities. 'Innovation' is the leading theme of the EU economic strategy to 2020 [14]. The European Union's Structural Funds, one third of total EU resources, have earmarked around 10% for support for research, both in people (funding for training and early careers) and facilites (such as 'science parks').

### European health research

'Health' is the term increasingly used for the field formerly known as 'medicine'. The World Health Organisation, in its 1948 founding articles, described health as 'a complete state of physical, mental and social well-being, and not merely the absence of disease or infirmity'. This raises the bar high, since most 'health' services are still primarily oriented to patients consulting with disease, and most healthcare resources are spent on citizens in their last year of life (and thus trajectory to death). Yet 'health' recognises the need to understand and respond to people on biological, social and psychological planes. If you define medicine to encompass these already - as some physicians and philosphers have done over the centuries - then there are grounds for retaining the word medicine. But issues of power have intruded. The authority of 'medical' doctors in defining and treating disease is challenged by other workforce disciplines 'allied' to medicine performing tasks for patients (nursing, caring), or who reject 'medicalisation' [15] of human experience. Similarly, there is a criticism of equating health with 'wellbeing' and 'happiness', which are unstable subjective measures, as though these are equivalent to 'disease' that is addressed by medical doctors.

The European Commission's fourth and fifth Research Framework Programmes included BIOMED 1 and 2 (1994-2002), which emphasised life sciences and basic biology, and gave some support for epidemiology. For the sixth Research Framework Programme, covering the years 2002-2006, there was a substantial shift [10]. With the development of new technologies of recombinant genetics, a high proportion of the biology and medical budget was directed towards genetics, while 'health' themes were relegated to a separate 'policy research' strand. For the seventh Research Framework Programme (2007-2013), the main focus has been on diseases (cancer, heart disease, respiratory disease etc) that match medical specialties and pharmaceutical approaches. The new paradigm is 'translational research', seeking to use existing, and develop new, knowledge to provide more effective treatments - and to 'translate' research into marketable and profit-making products. Nevertheless, as well as molecular and clinical research, the seventh Research Framework Programme has also a 'pillar' for public health, which includes health determinants and health systems research - although it receives only around 5% of the total research budget in the Health theme.

While there remain substantial bureaucratic obstacles for the reseacher to overcome in applying for funds, several structural changes have made accessing the European research programmes more attractive: the funds can now be used for all researchers including the work of those with tenured positions; there are mechanisms to draw on national co-funding; individual single-country science projects are now supported through the new European Research Council; and countries across the world are able to participate if they contribute to the project.

### Global health research

While the term 'health research' is mostly used today to include laboratory, clinical and population-level research, there is inconsistency. The Global Forum for Health Research, set up with support of the World Health Organisation following the landmark report of the Commission on Health Research for Development [16], has revised the term to 'research for health' in an attempt to emphasise public-health concerns for the population-level determinants of disease, as well as treatment [17]. Equally, there is growing recognition of 'health research systems', the organisational, social and economic frameworks that support health research. Funding of research in low and middle income countries led by the Gates Foundation for treatment of HIV, TB and malaria has come sharply up against the importance of healthcare delivery, access and uptake research to maximise success of laboratory-to-bedside programmes. And the contribution of prevention in reducing the global burden of diseases is recognised in the emerging agenda for chronic diseases research [18].

Historically, health research in low and middle income countries has been a mix of national and international programmes. The USA (for example, the Fogarty International Centre at the US National Institutes of Health) and European countries individually have been donors, sometimes tied to specific research institutes [19]. Since the report of the Commission for Health Research for Development [16], WHO has encouraged its member states to develop national health research strategies and programmes. The response has been patchy, as indicated by the limited number of countries with full descriptions on the website of the Council on Health Research for Development [20], but thriving indigenous research is expected to increase relevant research, to support researchers fostering the next generation, and to reduce the brain drain to western countries.

The European Commission had collaboration with 'third countries and international organisations' in its research programmes since 1994. This capability was included in the thematic programmes (health, food, IT etc) in the seventh Framework Research Programme (FP7). At the same time, the rules of FP7 were widened to allow applications, not just as partners but also as leaders, from almost all countries in the world, and for a focusing of calls on regions and across themes. For 2010, the FP7 programme brought together an 'Africa' research call from research topics (and funding) within the themes of agriculture, food and transport as well as health. And the instrument of 'ERA-nets', networks of national research organisations, can help researchers join together in planning research and feed ideas into the European programmes.

It may seem that there has been a slow European awareness of the needs for global health research. The torch for collaboration was kept in earlier years by a few countries in a semi-postcolonial way, with research programmes determined by the donor country, and the lack of technology infrastructures as well as financial attractions have led laboratory scientists to migrate to western countries. Nevertheless, the conjunction of the Report of the Commission on Health Research for Development, the financial resources of the Gates Foundation and the international concern on millenium development goals changed the situation markedly. The new agenda of globalisation brings new players to the table and alters the dynamics of priorities, incentives and practice [17].

There have also been important impacts and changes in direction. Beyond trials and marketing of pharmaceuticals, there is now recognition of research on delivery systems, health cultures and behaviours including uptake, and wider determinants. The trials of low technologies such as bed nets, and economic incentives such as micro-payments, are changing the paradigm of health research, bringing in local communities, requiring different governance and seeking different end points [21].

The European Union's economic and research policies are oriented towards innovation in support of economic development. EU support for health research emphasises biomedicine and technology, but there is less support for public health and health systems research. Two case-study examples in relation to globalisation are given below, and the Discussion considers three themes arising - contrasting research for private and public gain, the role of civil society organisations, and perspectives of social sciences.

## Case-studies

### Globalisation and Health at the European Commission

The European Commission Global Health Conference, held in Brussels in June 2010 [22] brought together three of the Commission's directorates with overlapping interests - the Directorate General (DG) for Health, DG Development and DG Research. These are not large spending directorates: two thirds of European Commission's annual funding of €141 bn are spent on the Common Agricultural Policy and the Regional Funds (which are directed towards the poorer countries and regions of Europe) [23]. DG Research has €7.5 bn (5% of the European Union's budget), DG Development €3 bn (2%) for direct overseas aid, and the DG Health and Consumers' budget, at €50 million, is just 0.1% of the whole total budget. The seventh Framework Research Programme allows applications from countries around the world when the researchers are collaborating with Europe.

The Conference had two days, of which the first was identified as technical and the second political. This reflected the structure of inter-governmental conferences such as the recent UN Climate Change conference, with initial work leading to final political declarations. Participants, up to the 400-person capacity of the European Commission's Brussels Charlemagne building hall, were invited through European representative organisations rather than member states alone. The opening sessions on health and development were given contemporary political emphasis with the words 'inequalities' and 'rights', although these were concerned more with moral debates than with practical and political questions of how to achieve balanced global economic development and thereby greater health for all. There was discussion on broad health issues, including workforce, communicable diseases and non-communicable diseases. Country-led international health strategies were presented, and the policies and programmes of the European Commission. Yet research was considered particularly from the paradigm of commercialisation by European pharmaceuticals manufacturers, and the protection of intellectual property. Of two workshops devoted to health, all six speakers in the workshop session 'Innovation' took this approach, explicitly promoting research for industry [22].

### Europe-India health research

Two examples of European collaboration with India on research in relation to health are considered. The Euro-India Research Centre (EIRC) has been established as "an information service to facilitate collaboration between Indian and European organisations (from industry and academia) for conducting joint RT&D through FP7" [24]. This coordinating support includes a National Contact Point service for liaison on specific research fields and calls as well as liaison for project implementation. Since 2007, there have been more than 140 partners in successful FP7 proposals, including 20 for the health calls. However, within the health projects in 2007, and despite the profound needs fof India, public health research was given very little precedence: 17 were for biomedicine, 2 were for health financing and one was a generic support network. In the Science in Society call, one of the four successful projects was for health - about patent protection in the pharmaceutical industry.

New INDIGO, an FP7 project led by the French national research agency CNRS, seeks to promote scientific collaboration and access to the European Research Area [25]. While providing a service across all scientific areas, New INDIGO chose to make its first call for funding of networking projects to start in 2010 in the field of 'Biotechnology and Health'. In this call, the three fields specified for proposals were all laboratory sciences - biomarkers and diagnostics, bioinformatics, and structural biology. Indeed, to emphasise the priority for industry research, New INDIGO web page noted as 'Important' on its 'News' a Flagship Mission to India for biotech SMEs (small and medium enterprises). The event is advertised as 'an opportunity to enter one of the world's fastest emerging biotech markets', from May 30th to June 4th 2010 in Bangalore, where 'EU participants will benefit from podium presentations to a selected audience of Indian public and private business and research organisations; [and a] customised schedule of one-on-one business meetings with pre-screened Indian potential partners, agents, distributors, licensees, and retailers' [25].

## Discussion

Globalisation is the new framework for understanding economic and commercial development, for addressing issues of environmental sustainability, for security and social justice. Health and research are part of this agenda, but what science is needed?

### Research for private and public gain

The European Commission's Globalisation and Health conference [22] was framed around European Union's policies and practices - spreading European influence by 'soft' means of discussion, exchange and funding, rather than 'hard' means of trade and war. The conference included participants expected to be critics, in the forms of NGOs and academics, as well as politicians. But the research theme debate left unresolved the crucial choices between international research for the private sector and for the public sector, and thereby the balance between research for medicine and research for health.

The Europe 2020 strategy [12] proposes a 'knowledge-based economy' through research and innovation for sustainable development. The policy of national research budgets growing to 3% of GDP is also maintained, with a continued emphasis on research to be funded by industry. DG Research has put effort into linking so-called small and medium enterprises (SMEs) with the publicly-funded research programmes, hoping to create synergy and expansion: an example is SMEs-Go-Health [26], a coordinating organisation providing support for "research-intensive, high technology SMEs" to join research consortia. Yet most SMEs, by the EU definition employing fewer 250 people, are usually without any research capability. Sometimes they can access research organisations providing services to small companies, but the research is mainly 'near product'. The strategy also encourages the protection of intellectual property through patents - away from a traditional European humanistic view that knowledge is universal. And experience is mounting (anecdotally) within DG Research of SMEs involved in research consortia that do well in the first year of the project but fail in the second - a feature much less common in public sector research.

There is an increased pressure to invest in technological research, and for companies to gain financial return in sales through the health care market. Yet healthcare systems are publicly regulated and paternalistic, and 'trade' is at cost to the public as payers of health insurance and taxes. Equally, the emphasis on laboratory research gives less value to social, behavioural and organisational research. The emphasis on developing effective medical interventions has led to a new paradigm of 'translational' research, which seeks to link the 'laboratory' to the 'bedside'. And this paradigm is increasingly driven by commercial interests. It is difficult to introduce the idea that the determinants of health lie outside the laboratory, in the wider aspects of society and economy, and that 'translational' research on effective interventions in this wider public-health field is as relevant to the health sector as narrower clinical research [27].

The pharmaceutical industry uses developmental work extensively, with a paradigm of steps from laboratory to human clinical trials (phase 1 to phase 4 trials) now enshrined by regulating agencies. By contrast, public-health innovation actions have no strategic framework equivalent to pharmaceutical research. They are usually described as 'projects', often one-off, context-specific, isolated from other equivalent work, without replication or scale-up, and perhaps weakly evaluated (including lack of economic evaluation). Prospective observational epidemiological research is funded, but large public health intervention studies are rare. As a result, regulatory agencies have limited evidence to promote effective public-health interventions, and also not able to reject those which are ineffective. Innovations in disease prevention and health promotion develop independently in European countries, with less joint learning and with resulting waste of resources.

The argument here is two-fold. First, that within medical research there should be greater emphasis on public health and health systems research - and a reduction in investment on pharmaceuticals research - because the health gain will be greater. There is a social benfit from not-for-profit, or non-patentable, research. Second, social and services innovation should be recongised to be as beneficial as for-profit, patentable research. There are physical technologies and there are social technologies: disease treatment may use physical treatments while disease prevention can use social and behavioural interventions. As well as recognising the need for innovation for both business and services "in all sectors, including the public sector", the European Commission proposes "new ways of meeting social needs which are not adequately met by the market or the public sector" [14].

The health challenge of globalisation is how to succeed within the wider for-profit market system. Corporate capitalism seeks not just to be within a market, but to control it [28]. If research and innovation are the basis for commercial success, capitalism will seek to control and direct them towards corporate rather than public benefit. The European Union has policies for innovation which are stated to address social as well as economic issues. However, the meaning of social may be 'more and better jobs and increased social cohesion', that is employment protection, rather than broader actions for the benefit of society as a whole.

The returns from research and innovation, and their implementation in health and healthcare systems, should be calculated and set against the costs of alternatives. The pharmaceutical industry is closely linked to the major global donor in the health field, the Gates Foundation, promoting the paradigm of treatment for diseases (HIV, TB, malaria) that are also preventable by alternative social public strategies and investment. Funds go into treatment of patients now while further cases arise, a 'downstream' policy which perpertuates the disease and thus the response. Thus, while programmes for drug treatment of HIV have been rolled out with the strong support of industry, the Global HIV Prevention Group [29] have estimated that scale-up of existing prevention tools would lower the incidence of HIV by nearly two-thirds by 2015. Since the total research capacity is limited, economics should compare investment in public-health research in competition with, rather than in addition to, pharmaceutical research. Health research can provide a balance in approaches and to deliver sufficient evidence to influence policy and practice in more socially beneficial ways.

### Civil society

One contribution to balance research to benefit industry can come through civil society organisations (CSOs). There is a growing literature on public involvement in health research in low-and middle-income countries [30]. Areas of involvement have included developing the research agenda, design, methods and impacts. Studies report benefits - and difficulties - for researchers, research participants and community organisations; but there is little research published on the impact of public involvement on research funding and commissioning. Yet civil society organisations are interested also in the systems of health research. In STEPS [31], funded by the Science in Society theme of FP7, CSOs in the twelve EU new member states have organised workshop meetings with researchers and national health research commissioners. The sessions showed a strong interest from the CSO participants: as well as applying knowledge passed from others to be implemented as practice, they also see themselves promoting research themes and being part of the research development process.

In most European health research with civil society involvement, the focus has been patients rather than the public [32]. In a study of the UK health research system, Hanney et al [[33]; p9] comment: 'Organised patient groups tend to push for more research in their particular fields, and the lack of a strong advocacy group for public health may have contributed to the traditionally low levels of funding in that area'. For example, in the field of rare diseases, the pharmaceutical industry has been assiduous in promoting, and indeed often rewarding, patient involvement: the 'European Patients Forum' is almost fully funded by six pharmaceutical companies [34].

But engagement of civil society organisations is also promoted by the Global Forum for Health Research. In 2010, working with the People's Health Movement, a call was made for research proposals from civil society organizations [35]. CSOs were seen as participants in the entire research process, from design through to dissemination, and could contribute to proposing interventions and evaluation methods, as well as influencing policy choices and uptake of research into practice. There were 93 proposals received, from 53 countries and across 5 languages. Four selected research proposals are to be supported with mentoring, networking and a cash award of up to USD 10,000. This initiative begins to balance the involvement of for-profit industry in low and middle income countries.

### Social science perspectives

Public health, which brings social sciences into dialogue with bio-medical sciences, has to argue its case for action. Epidemiology is able to demonstrate risks and associations quantitatively, and to monitor and demonstrate impacts from interventions. In much public health science where the randomised controlled trial is difficult to apply, methods are often descriptive and inferences of risks and benefits have to be considered through non-experimental criteria [36]. Yet even where a well-conducted trial has shown compelling benefit, for example, in prevention of neural tube defects with folates, policy-makers may delay public programmes [37].

Surveying the public health research systems in European member states, the lack of development of social sciences for health research was evident [38]: the main recipients of national research funds were the traditional science academies, while the ministries of health funded public-health institutes mainly undertaking laboratory and sanitation sciences. In western European countries, social sciences have developed within universities, producing both quantitative and qualitative research, and linking to health services research, health promotion and health economics. These social science inputs complement medical science and practice in public-health: research needs to address both social and biological determinants of disease, and the effectiveness, efficiency and equity of the health system.

How does the emphasis, in the Lisbon Agenda, on science for innovation by the commercial sector match the needs of health research at European and global levels [39]? Steiglitz [40] and Chen et al [41] developed the case for both knowledge and health as 'global public goods' in a colloquium by the United Nations Development Project. The challenge to health has come in the past decade through pressure from global pharmaceutical companies to maintain profits in the face of international concern for access to drugs [42]. The World Health Organisation's so-called 'Global strategy and plan of action on public health, innovation and intellectual property' is only passingly about public health and very much about intellectual property protection. But the European discourse can be broader: for example, the European Commission Research Directorate's 'Science in Society' programme [43] has proposed a balance between an approach valuing 'economy', with technological solutions of social problems and a passive (consumer's) role of civil society, compared with research valuing 'collectivity', with more low-tech and social innovations, unrestricted transfer and use of knowledge (while supporting traceability of their origin and influence), and emphasis on public accountability and utility. This should have resonance in globalisation and health debates.

In the global context, there is a need for a vision of what future policies and infrastructures for health research should be. An interdisciplinary mix of skills is required; teams that have flexibility and sufficient skills to tackle both short and long-term questions; ability to learn from and contribute to international experience; capacities for the staff to retain their career trajectories and respond to changing policy and research priorities. At the same time, there should be programmes and funding which encourages this research, with a stature equivalent to the biological and technical sciences. Public health combines medical and social sciences, and public-health research is disseminated through international publications, meetings, media and the internet. The European Union, as well as national and international programmes, must give more support to public health research, and its standing in the global research market, for it to be able to contribute fully to society.

## Conclusions

For many centuries, global knowledge transfer has been an important driver of cultural and economic development. The European Commission is promoting science for innovation both internally in European Union member states and also through international transfer of people and ideas. In the health field, the dominant bio-medical model for research links innovation with pharmaceutical research for profit. A second paradigm, of social science for economic benefit, is particularly relevant for global health. Further support is needed for policies and partners, including civil society, to redress the current emphasis on biotechnology research, aimed at treatment, and to develop social sciences for prevention and public health.

## Competing interests

The author declares that they have no competing interests.

## Authors' information

MM has worked and undertaken research in public health in UK and for international organisations (WHO, European Commission). In collaboration with the European Public Health Association, he has contributed to describing and supporting public-health research in Europe. He was invited to contribute from this perspective to the UK/India workshop 'Health systems, health economies and globalisation: social science perspectives' held at the London School of Economics in July 2010.
